# “Death as the One Great Certainty”: ethical implications of children with irreversible cardiorespiratory failure and dependence on extracorporeal membrane oxygenation

**DOI:** 10.3389/fped.2023.1325207

**Published:** 2024-01-11

**Authors:** Katie M. Moynihan, Lisa S. Taylor, Bryan Siegel, Natasha Nassar, Efrat Lelkes, Wynne Morrison

**Affiliations:** ^1^ Department of Pediatrics, Harvard Medical School, Boston, MA, United States; ^2^ Department of Cardiology, Boston Children’s Hospital, Boston, MA, United States; ^3^ Children’s Hospital at Westmead Clinical School, Faculty of Medicine and Health, The University of Sydney, Sydney, NSW, Australia; ^4^ Office of Ethics, Boston Children’s Hospital, Boston, MA, United States; ^5^ Clinical and Population Translational Health, Children’s Hospital at Westmead Clinical School, Faculty of Medicine and Health, University of Sydney, Sydney, NSW, Australia; ^6^ Department of Pediatrics, MaineGeneral Medical Center, Augusta, ME, United States; ^7^ Department of Anesthesiology and Critical Care, Perelman School of Medicine at the University of Pennsylvania, Philadelphia, PA, United States; ^8^ Divisions of Critical Care and Palliative Care, The Children’s Hospital of Philadelphia, Philadelphia, PA, United States

**Keywords:** extracorporeal membrane oxygenation (ECMO), ethics, communication, death & dying, pediatric

## Abstract

**Introduction:**

Advances in medical technology have led to both clinical and philosophical challenges in defining death. Highly publicized cases have occurred when families or communities challenge a determination of death by the irreversible cessation of neurologic function (brain death). Parallels can be drawn in cases where an irreversible cessation of cardiopulmonary function exists, in which cases patients are supported by extracorporeal cardiopulmonary support, such as extracorporeal membrane oxygenation (ECMO).

**Analysis:**

Two cases and an ethical analysis are presented which compare and contrast contested neurologic determinations of death and refusal to accept the irreversibility of an imminent death by cardiopulmonary standards. Ambiguities in the Uniform Determination of Death Act are highlighted, as it can be clear, when supported by ECMO, that a patient could have suffered the irreversible cessation of cardiopulmonary function yet still be alive (e.g., responsive and interactive). Parallel challenges with communication with families around the limits of medical technology are discussed.

**Discussion:**

Cases that lead to conflict around the removal of technology considered not clinically beneficial are likely to increase. Reframing our goals when death is inevitable is important for both families and the medical team. Building relationships and trust between all parties will help families and teams navigate these situations. All parties may require support for moral distress. Suggested approaches are discussed.

“Death is the one great certainty. The subject of powerful social and religious rituals and moving literature, it is contemplated by philosophers, probed by biologists and combated by physician”. Morris B. Abram 1981 (1)

“If one subject in health law and bioethics can be said to be at once well settled and persistently unresolved, it is how to determine that death has occurred”. Alexander Capron 2001 (2)

## Introduction: ambiguity defining death—lessons from death by neurological criteria and parallels for extracorporeal membrane oxygenation

While death is “the one great certainty” of humanity, controversies exist surrounding accepted medical standards to define death (1–6). Biologically, somatic death is a gradual process whereby entropy overwhelms homeostasis as tissues variably endure oxygen deprivation (7). But rather than the state of isolated cells, societal death relates to the fate of a “person,” and “certainty that the process has become irreversible” has much greater clinical relevance (8, 9). The World Medical Assembly and the Uniform Determination of Death Act (UDDA) define death as having occurred when: “*an individual … has sustained either (1) irreversible cessation of circulatory and respiratory functions, or (2) irreversible cessation of all functions of the entire brain, including the brain stem*” (4, 8, 9). Death by cardiopulmonary criteria is widely accepted due to the obvious bodily changes which occur after cessation of circulation. It is assumed that, when circulatory death occurs, neurological death will follow. Declaring death by neurologic criteria (DNC) can lead to controversy as the reverse is not always immediately true; some physiological processes, such as cardiac function, can continue if ventilator support is maintained. Numerous authors have argued that DNC is a social construct or legal fiction not synonymous with human death (3–6, 10–13), and some US states allow conscientious objection to DNC (14). Notwithstanding these and other challenges, empiric establishment of uniform standards of DNC (4, 13, 15) provided a societal definition for death of an individual which (1) offered closure for families, (2) provided a path to discontinue non-beneficial care to prevent overwhelming healthcare systems, (3) afforded legal protection for physicians, and (4) facilitated organ donation.

Extracorporeal Membrane Oxygenation (ECMO) is a highly technologic, invasive intervention that is often emergently instituted to temporarily replace pulmonary and cardiac function. Thus, this technology could contradict strict interpretation of the UDDA definition that complete, irreversible failure of the native heart or respiratory system functions constitutes death. A person who still has neurologic function (even if heavily sedated) is clearly neither intuitively or clinically dead, highlighting the challenges of the UDDA definition in light of modern technology (16–18). Though death during the hospitalization is certain as indefinite support is not feasible, arguably despite irreversible cardiorespiratory failure they are living on ECMO (19)—essential processes are maintained by a machine. The most recent update to the DNC Consensus Guideline has begun to acknowledge that some UDDA language may need updating or clarification, noting in the terminology section that they choose to use “permanence” rather than “irreversibility” in their language, and adding that medical interventions will not be used to attempt restoration of function (20).

In this paper, we describe and analyze cases where ECMO raises ethical issues after a disease process has led to total, irreversible loss of patient cardiopulmonary function and draw parallels with the evolution of DNC definitions (21). Both sets of circumstances highlight the difficulties—in both terminology and societal consensus—that occur with defining death as our technologic ability to support patients expands. ECMO support will inevitably result in some cases where the only thing ECMO achieves is delaying death, so considering such cases will be important in clinical care (16). We argue that lessons learned from DNC may apply in these ECMO cases and warrant a stakeholder-approved approach to exploring the novel use of therapies in children such as ECMO in the setting of irreversible cardiorespiratory failure that cannot be managed by means such as transplant or assist devices.

### Case 1: alive despite irreversible heart death^1^

Baby TL was born with complex congenital heart disease. The blood supply to the heart relied entirely on pressure within a hypoplastic, dysfunctional right ventricle chamber. Survival was possible only with heart transplant, so he was listed immediately for cardiac transplantation. He deteriorated despite exhaustive attempts to medically manage his challenging circulation, and was emergently cannulated for extracorporeal membrane oxygenation (ECMO) for cardio-respiratory failure. Immediately following ECMO initiation, the right ventricle chamber was decompressed resulting in inadequate coronary blood flow, thus his heart infarcted. Baby TL remained asystolic with no cardiac electrical activity or pulsatility, i.e irreversible heart death, with no chance of recovery. The ECMO machine maintained circulation to his body, so he was warm and initiated some breaths on the ventilator, but required high doses of sedatives and was minimally interactive. The initial goal for ECMO was as a bridge to heart transplant, but he suffered kidney injury and was no longer a transplant candidate. Thus, baby TL had irreversible, permanent circulatory failure.

### Case 2: alive despite irreversible lung failure

SB was a 16 year old girl with cystic fibrosis diagnosed in infancy. In adolescence, she developed progressive pancreatic insufficiency and did not adhere to medications, often presenting with diabetic ketoacidosis and developing significant malnutrition. SB also had recurrent pulmonary infections requiring frequent hospitalizations, and her pulmonary function tests indicated severe disease. A viral infection led to endotracheal intubation. With worsening hypoxemia and hypercarbia, she was urgently cannulated onto ECMO. Because of prior non-adherence and poor nutritional status, SB was deemed to not be a lung transplant candidate. Attempts to reduce sedation failed with symptomatic breathlessness despite continued invasive ventilation, although SB was occasionally able to interact with her family and friends. She continued to require ECMO for refractory hypoxemia, with no ability to wean support and no recovery seen after six weeks. Thus, SB had irreversible, permanent respiratory failure.

## Ethical analysis

Many challenges associated with ECMO discontinuation for children with irreversible cardio-respiratory failure and no destination options parallel those in unilateral decision-making in DNC (Table 1). In this section we discuss how, although there is ethical justification for withdrawing non-beneficial ECMO support when all stakeholders are in agreement, if surrogates insist on continuation or value continued biological life, discontinuation of support over their objection is more ethically complex.

**Table 1:** Parallels between ECMO and ambiguity surrounding death by neurological criteria.

Technology staving off “death” in the traditional sense	Patients supported on ECMO may have sustained irreversible, permanent cessation of native circulatory and respiratory function, yet oxygenation at a cellular level is maintained by technology. Does ECMO support to maintain physiological functions in the setting of irreversible cardio-respiratory failure without potential for recovery, transplant or alternate destination device support challenge UDDA definitions in a similar fashion to patients meeting brain-death criteria sustained with ventilator support? (21) As some have argued for death by neurological criteria, could these patients be considered “as good as dead”? (22)
Maintained Physiological Processes	In stark contrast to the classic picture of circulatory death with long historical precedent portrayed by the media as a cold, blue, pulseless immobile corpse, patients meeting clinical brain death criteria look “alive”, with beating hearts and warm bodies that, though fully technologically dependent, continue physiologic processes including menstruation and pregnancy (5, 11, 12). In parallel, patients supported on ECMO, despite irreversible cardiorespiratory failure, also may appear warm and pink with physiological functioning.
Resource constraints are at the forefront	ECMO is undeniably resource intensive (23). The concept of brain death originally evolved in response to the advent of mechanical ventilation and contemporary improvements in resuscitation in the 1960s with associated concerns relating to prolonged nonbeneficial care and the risk of irrecoverable patients filling hospital beds (4, 13). There is again concern that healthcare resources are limited, amplified by the complexity of ECMO (24, 25). In both paradigms, innovation has driven medical and technological advances that facilitate prolonged support in populations previously considered unsuitable (26–31). ECMO is highly resource intensive engendering competing stewardship obligations, fair allocation considerations, and risk of moral injury for teams in providing what they perceive as potentially non-clinically beneficial or inappropriate care (32, 33).
Complex terminology and societal awareness	The concepts, definitions and technology for ECMO and DNC are complex and medicalized, and are challenging to comprehend and operationalize for both healthcare providers and the lay population (34, 35). Particularly with COVID-19, general knowledge of ECMO has risen although the quality and accuracy of information is variable (36).
Subjectivity and Competing interests	Clinicians have a fiduciary responsibility to avoid harm, yet also to respect varying views, preferences and values. Both ECMO and life sustaining therapy following determination of DNC are invasive with substantial clinical burdens, yet weighing benefits and harms is value dependent and highly subjective (18). Interviews with bereaved parents suggest they may not perceive the same burdens (37).
Uncertainty and “Chronic” ECMO	ECMO use is expanding with long runs becoming more routine with continued innovation. Currently use is restricted to the ICU—there is no destination therapy, yet history has shown that technology once deemed extraordinary may become customary. Cases of prolonged ECMO support are reported in the literature and miniaturized devices suitable for home or longer-term ECMO therapy may be possible in the future (28, 29). Long-term ECMO use raises similar questions to DNC, regarding decisional authority and equitable distribution of limited, infrequently utilized, resource-intense therapies (26, 32, 38–41).
Removal of technology	While patients eventually become unsupportable, organ-sustaining therapies often need to be removed to eventually allow either cardiopulmonary or neurologic death in the traditional sense. While much has been written on the ethical equivalence of withdrawing vs. withholding life-sustaining therapies, in a practical sense it feels harder to actively stop an instituted therapy even if no longer clinically indicated (42). It requires a burdensome decision and act and presents the additional challenge of sociocultural and public scrutiny, reminiscent of recent high-profile DNC cases, given the legal and ethical deference afforded to surrogates in western medicine when decisions are arguably values-based (3, 5, 43, 44). Thus, without defined limits, parents who value continued biological life may demand that ECMO continue, even when ECMO will only forestall an imminent death, similar to continued ventilation in DNC.
Response to lack of guidance	As clinicians continue to engage in experimental and long shot treatments, prognostic uncertainty is increasing without a data-driven decision-support framework to inform ECMO decisions (26, 32). Despite challenges, specific processes are recommended for determining “brain death” on ECMO (45, 46). Beyond this, guidance is lacking as to when a patient has met the criteria for cardiopulmonary “death” in the absence of an exit strategy to recovery, device or transplant (19). Notwithstanding, even with guidance, those with vitalist values may demand continued ECMO support, similar to conscientious objection to DNC.

Traditionally, there are four potential goals for ECMO use: as a bridge to recovery, device, or transplant, or as a bridge to decision regarding one of these alternatives (47–49). Outside these parameters, continuing ECMO leads to death in the ICU within days to months, so is felt to be a prolongation of support that has no clinically beneficial outcome (16, 19, 26, 47, 50, 51). In current practice, with no long-term or home “destination” therapies yet available, it has often been referred to as a “bridge to nowhere” (21, 52–55). Defining acceptable ECMO use in children with irreversible cardio-respiratory failure and no bridging options is challenging at baseline with limited published guidance (26, 32), but is especially difficult when patients are neurologically intact (16, 17, 19, 56). As a pluralistic society, there are multiple ethically reasonable ways to approach a given situation. Recent ethical analyses focus on withdrawal of ECMO in awake adolescents/adults over their dissent which is uncommon in pediatric ECMO (16–19, 56). A comprehensive exploration of pediatric ethical issues using varying ethical frameworks and lenses is critical to ensure that rationales for decisions are robustly constructed (17, 18, 23, 55–57).

There are strong ethical arguments that clincians should not initiate or continue medically inappropriate therapies (33, 51, 56). However discontinuing technology is especially challenging in pediatrics, when it involves overriding patient/surrogate/parent's objections (50). Parental decision-making is generally given the utmost respect, unless the decisions are harming the child (18). Whether (and how much) ongoing, highly invasive support that only prolongs the dying process causes harm to the child is an important question and may be case-specific (58). ECMO support is often emergently initiated without time for fully informed conversations with a decision-maker (59) based on clinician judgment as to whether ECMO has a chance of leading to a good outcome. ECMO is a high-risk, high-cost, high-reward therapy at the intersection of standard ICU care and innovative therapy. So, if clinicians prognosticate with a high degree of certainty that the patient's organs will not recover, and the patient is not a transplant candidate, can prolonging death in this way be considered beneficial? For some, delaying death, even for a short while, may have value (a consequentialist, vitalist argument). A patient, or surrogate, may express a desire to be maintained on ECMO, and it may be physiologically feasible. Does a demand for a therapy to avoid death outweigh all else? The relative weight of benefits vs. burdens and what constitutes a good outcome or best interests are highly subjective (60, 61). Clinicians and surrogates may legitimately have different views of what is in the best interests of the patient (62, 63). The best interest standard is also applied differently internationally; in the US, clinicians typically override parental authority only if parental decisions cause substantial/significant harm to the child, while in some countries medical authority is given more priority (62, 64, 65). Resource limitations, however, may change this calculus as the impacts on other potential patients or the healthcare system affect what it is appropriate to provide for any individual (23). When death is unavoidable and proximate, does discontinuation of circulatory support fall within the zone of parental discretion or harm? (17, 56, 58, 66).

There are other ethical frameworks/reasoning that support clinician and institutional interests in establishing standardized indications for ECMO cannulation/discontinuation (18, 56). If the compassionate act, when death is inevitable, is to take the burden of end of life decision-making away from a family, the virtues of discernment, altruism, beneficence, honesty and integrity might justify allowing the medical team to determine that decannulation is appropriate. One could also consider the professional duties of a physician through a deontological model. It would not be possible, or desirable, to support every dying child with invasive technology to gain limited additional time, so the categorical imperative would argue that there is no moral obligation to continue ECMO when the therapy is not clinically beneficial. This also aligns with the professional medical ethics model of decision making (51, 67, 68). There is also a duty to be truthful—regarding prognosis, imminent death and false hope, especially in cases with little uncertainty about the outcome, such as with irreversible and permanent dependence on ECMO. Unfortunately, medical certainty does not always lead to trust in authority, and fear of litigation, social media responses, and ratings of physicians and hospitals have created a culture in which some clinicians are conflict averse. Healthcare professionals also have a duty to society. The extensive resources utilized in ECMO—beds, personnel, blood, medication, finances—are limited (23). ECMO is also a therapy with inequitable access (69, 70). When perceived to be not clinically beneficial, when death is imminent, continued utilization of ECMO then becomes unjust in a society where healthcare is a finite resource (23, 26). Providers have stewardship obligations—to be judicious in the use of these therapies, and thus discontinue ECMO when death is inevitable (26). Reductionists would reason, when death is inevitable, it should be respected as a biological truth. Judicious use of ECMO under standard care conditions in this way differs from resource limitation settings (or “crisis” standards of care) when the ethical weight of individual benefit vs. population utility shifts, requiring specific processes for procedural fairness (71).

In contrast to frameworks that support discontinuation of ECMO despite parental wishes, other approaches center on deference to parents or other surrogate decision-makers in deciding if and when these supports should be discontinued. As long as a patient is not suffering or being substantially harmed (highly subjective determinations) (72), a care ethics perspective suggests that the relationship between parent and child is sacred and that parents are uniquely situated to determine what is best for their child (73). Thus, even if the medical team determines that continuation of ECMO is inappropriate, the family may have a moral argument for continuing (74). Virtue ethics also requires humility; clinical judgment can be imprecise and flawed, and hubris in circumstances of uncertainty can damage the therapeutic alliance. In these situations, narrative ethics—understanding the patient/family's story, understanding *how* we arrived at this scenario, and similarly giving room to the narrative of all stakeholders—may mitigate some of the friction and moral distress. If the medical team does proceed with removal of ECMO, narrative ethics may provide a basis for much-needed discourse and support of the family. Contemporary medical advances mean it is also possible to be awake, interactive, and walking on ECMO which could be reasonably perceived as quality of life. But what if the only purpose of ECMO is sustaining perfusion without these other benefits? Vitalist values argue that mere biological function should be respected, and further complexity arises when patients, families or team members perceive any life to be of value in spite of dependence on technological support (19).

In all scenarios in the care of a patient with irreversible cardiorespiratory failure and permanent dependence on ECMO, *all* ethical frameworks are relevant; none are dispositive (50). Further, as with DNC, unilateral decision-making around ECMO over parents' values-based objections may be ethically fraught, warranting collaborative communication and shared decision-making approaches (75).

## Implications

Just as opposition to DNC has evolved, we anticipate objections to ECMO discontinuation in children when used outside the four “bridges” to increase in the future. In this section we offer actionable practice changes. Ideally preemptive conversations both within teams and with patients/families can avoid conflict (57). Such discussions may help avoid escalating to advanced technologies in cases where there is little hope of benefit. However now more than ever there is abundant misinformation and unrealistic media portrayals as well as great scrutiny on medical decision-making and societal mistrust (34–36). Unjustified bias in healthcare decisions is well described, requiring medical providers to endeavor to re-establish societal trust in decision-making as well as advocate for accurate media representation (76–78). At the same time we are at a moment of great distress within the medical profession, with an epidemic of burnout, mental health crises, and colleagues leaving medicine (79). While rebuilding societal trust, we must also support our teams. Variability in practice and frequent conflict suggests no all-encompassing principle or theory will resolve all cases (50) and much ongoing collaborative, conceptual work is required to forge a path toward defining the appropriate use of ECMO in children. The goal is to avoid relying on conclusory definitions of death or candidacy limitations that might have the benefit of expedience for providers but are in conflict with patient and family values while also avoiding simply continuing a resource intensive, invasive therapy due to fear of conflict (26, 27).

We need to reframe our language to make clear that we are not burdening a family with a life/death decision but rather supporting them through an impossibly challenging situation. Death is often seen as a failure in our culture and medical practice, and has become highly medicalized (80). If we ask patients or their surrogates to decide to discontinue life-sustaining therapies, it is akin to asking them to choose if they or their loved one will die. This framing of discontinuation as optional unfairly burdens our patients and their families, and may be harmful (43, 81–83). Though input from patients and their surrogates is important in end-of-life decision making, connoting that death is a result of that choice is a fallacy. Our communication has to change when faced with decisions between two tragic options. In these situations, more than most, words matter. Death is a result of the underlying disease process, not decisions of the medical team, patient or family. To state otherwise unjustly burdens decision-makers by unfairly suggesting that outcomes depend on the decision. In the literature and when we approach our patients and their surrogates to discuss therapies such as ECMO or ventilation we use the term life-sustaining therapy or “extracorporeal life support” indicating that our emphasis is on life and death. This term is flawed, and we would be more accurate to discuss organ-sustaining therapies as is applied for or organ-replacing therapies such as “renal replacement therapy”. A change in our language would potentially allow us to advance our conversations and avoid implying that patients, surrogates or patients/carers and clinicians are “choosing” death.

We need to reframe our focus from fighting death at all costs to encouraging acceptance when it is inevitable (on the part of patients, families, and healthcare teams) (80, 84). Death indeed is the one great certainty, yet in modern medicine, survival is expected (80). Our fixation with avoiding mortality, preoccupation with quantity rather than quality of time, and the need for certainty may fail those patients that die and their families (84). We live in a pluralistic society with differing values and perspectives as to what is a life worth living, as well as emphasis on respecting values that many would consider idiosyncratic. With contemporary critique of DNC, our consideration of somatic death also needs to be reframed in response to evolving technological developments. Complete consensus in defining life and death is not feasible. Framing what the extent of disease process means for the patient as a person may be enough, in most circumstances, to reach agreement regarding compassionate ECMO discontinuation (21, 57). Communication guides suggest using clear language (e.g., “die” rather than ambiguous terms such as “pass away”) and describing the child as dying in spite of the support to ensure clarity and encouraging honest and courageous conversations about what is or is not possible with ECMO (59). Life and death scenarios are commonplace in the ICU. Transparent, open communication with families and team members, trust and alliance with families, and listening and understanding their stories are all crucial elements. While parents generally desire equal medical team input in shared decision-making for big picture decisions, such communication should be personalized (57, 85, 86). Religious or cultural contexts may also be a strong influence on surrogate or parent/caregiver's conceptualization of technology discontinuation. For some families using strong medical recommendations and working with respected community leaders are key steps (43, 87). For others, particularly when there is distrust in the medical team and perceived unfair treatment, parents may want to retain decision-making authority and resist directive approaches—which makes addressing the trust issues a priority. Finally, one must consider the potential for disparities in who is offered ongoing time on ECMO, particularly with known institutional, regional and individual provider variability in ECMO support (88). It is essential to ensure processes are in place to mitigate any potential for bias, and research is also warranted to follow if differential approaches are used for populations with different levels of resources, education, representation.

Disagreements related to continued ECMO indication may be some of the hardest we currently face in the ICU. The toll on the family and on the medical team may be immense. In circumstances where values based conflict is anticipated, we ought to use all tools at our disposal as early as possible to avoid relational harm and reach an ethically supportable resolution that mitigates provider moral distress (50). Paticulary with misrepresentation with the media (34, 35), as with DNC public perception and understanding of invasive organ support at the end of life is challenging and consensus on strategies to mitigate harm are important. This includes involvement of trusted persons, primary teams, or religious authorities, and consultation of both ethics and of pediatric palliative care may be invaluable for the patient, family, and team (32, 89–92). Striving for alignment with patients and their family during this time is crucial, and managing disagreements without abandoning patients and families is key. We must proceed with integrity, maintain patient dignity, and aggressively treat symptoms at end of life. A thoughtful, compassionate, patient/family-centered approach to providing a “good death” with ECMO discontinuation is a moral imperative (57, 93, 94). Moral distress for the medical team should be anticipated and may be particularly heightened when caring for alert patients. Though withdrawal and withholding are considered ethically equivalent (42), for some, active participation in the death of a patient who can communicate may be contrary to their personal beliefs. Our duties then extend to supporting the medical team through conversation, ethics consultation and involvement of palliative care providers.

### Case conclusions

In both cases ECMO was discontinued after significant, lengthy conversations with the patients' families (in Case 2, the patient chose not to be involved in discussions), the patients' primary teams, the ICU teams, palliative care team and ethics consultants. Primary bedside nurses and family supports, which included spiritual care and extended families, were able to join multiple family meetings. The medical team approached discussions emphasizing that death was inevitable rather than a decision to be made. Proceeding with removal of ECMO support thus was presented as the recommended next step. Neither family dissented. Timing of discontinuation was a collaborative decision with the family, allowing for specific hopes—the baby to have his first Christmas, and the teenager to say goodbye to loved ones (59). Discontinuation of support was planned in detail to prepare the team and the family with child-life services, music therapy, and palliative care supporting the family, patient, and team throughout this process (93). For both patients, their deaths appeared peaceful.

## Conclusion

ECMO technologies can sustain physiological processes with a machine despite irreversible cardio-respiratory failure. Examining parallels with continued physiologic support after DNC and controversies that arise can be informative. We advocate that attention to semantics, societal education, and reframing the meaning of the disease for the individual child's life and therapy are needed. This requires proactive implementation of a systematic approach to decision-making. This will reduce decision-making burdens on families, minimize conflict around decannulation, enhance appropriate utilization of ECMO and intentionally focus on compassionate, value-centered, end of life care for patients on ECMO and their families.

## Data Availability

The original contributions presented in the study are included in the article/Supplementary Material, further inquiries can be directed to the corresponding author.

## Footnotes

^1^Details of both cases have been altered to preserve confidentiality.
